# Combined medial patellofemoral and medial patellotibial reconstruction for patellar instability: a PRISMA systematic review

**DOI:** 10.1186/s13018-020-02072-z

**Published:** 2020-11-12

**Authors:** Rocco Aicale, Nicola Maffulli

**Affiliations:** 1 Department of Musculoskeletal Disorders, Faculty of Medicine and Surgery, University of Salerno, 84084 Baronissi, Italy; 2 Clinica Ortopedica, Ospedale San Giovanni di Dio e Ruggi D’Aragona, 84131 Salerno, Italy; 3 Barts and the London School of Medicine and Dentistry, Centre for Sports and Exercise Medicine, Queen Mary University of London, Mile End Hospital, 275 Bancroft Road, London, E1 4DG England; 4 Faculty of Medicine, School of Pharmacy and Bioengineering, Guy Hilton Research Centre, Keele University, Thornburrow Drive, Hartshill, Stoke-on-Trent, ST4 7QB England

**Keywords:** MPTL, MPFL, Medial patellotibial ligament reconstruction, Medial patellofemoral ligament reconstruction, Patellar dislocation reconstruction

## Abstract

**Background:**

The medial patellofemoral ligament (MPFL) works in association with the medial patellotibial ligament (MPTL) and the medial patellomeniscal ligament (MPML) to impart stability to the patellofemoral joint. The anatomy and biomechanical characteristics of the MPFL have been well described but little is known about the MPTL and MPML. Several reconstruction procedures of the MPFL with semitendinosus, gracilis, patellar and quadriceps tendons, allografts and synthetic grafts have been described. No clear superiority of one surgical technique over another is evident.

**Methods:**

A systematic review of the literature was conducted using PRISMA guidelines. Inclusion criteria were articles that reported clinical outcomes of combined reconstruction of MPTL and MPFL. The methodological quality of the articles was determined using the modified Coleman Methodology Score (CMS).

**Results:**

Nine articles were included, reporting the clinical outcomes of 197 operated knees. The surgical procedures described include hamstrings grafting and transfer of the medial patellar and quadriceps tendons with or without bony procedures to reconstruct the MPTL in association with the MPFL. Overall, good and excellent outcomes were achieved. The median CMS is 70.6 ± 14.4 (range 38 to 84).

**Conclusion:**

Different techniques are reported, and outcomes are good with low rates of recurrence. The quality of the articles is variable, ranging from low to high. Appropriately powered randomized controlled trials are needed to better understand what the adequate indications for surgery in patients with patellar instability and clinical outcomes are. Combined reconstruction of MPFL and MPTL leads to favourable clinical outcomes, supporting its role as a valid surgical procedure for patellar stabilization.

## Introduction

Lateral patellar dislocation is a common cause of knee injuries and anterior knee pain, associated with haemarthrosis, especially in young patients [1, 2]. The patella is stabilized by ligaments, muscle and the trochlear groove [3]. The major stabilizing ligamentous structure, the medial patellofemoral ligament (MPFL), works in association with the medial patellotibial ligament (MPTL) and the medial patellomeniscal ligament (MPML) to impart stability to the patellofemoral (PF) joint [4]. The MPFL is considered the major medial restrictor while the others are secondary [5]. Patella alta, a large Q angle, a hypoplastic lateral femoral condyle and congenital ligamentous laxity are associated to recurrent patellar dislocation [6]. The MPTL and MPML contribute to limit the lateral translation of the patella, and this contribution increases from 26% in extension to 46% at 90° of flexion [1].

The anatomy [7], imaging [8, 9] and biomechanical characteristics [1, 5] of the MPFL have been well described, but little is known about the MPTL and MPML [1, 5, 8–10]. The MPTL is located 13.7 mm distal to the joint line and 3.6 mm proximal to the distal border of the patella, 9.4 mm distal to the joint line and in line with the medial border of the medial tibial spine [11]. When choosing a graft for reconstruction, it must be considered that the MPTL is stiffer than the MPFL. The MPTL and MPML, though considered secondary restrictors, have an important role in maintaining joint stability, especially in the final phases of extension, opposing the lateral traction of the quadriceps [12, 13].

The choice of technique to restore the stability of the PF joint needs to consider the skeletal maturity of the patient to avoid injury to the distal femoral physis [14–16]. The recommended treatment for recurrent patellar dislocation, in patients with normal bony morphology, is ligamentous reconstruction [17]. This usually involves MPFL reconstruction with the addition of procedures that improve, in specific cases, the alignment and the congruence of the patellofemoral joint [18], although isolated MPFL reconstruction produces good results [19–21]. Furthermore, good results with low complication rates are obtained with combined MPTL and MPFL reconstruction [22–24]. To the best of our knowledge, no studies compared outcomes of isolated reconstruction of the MPFL versus isolated reconstruction of the MPFL, or combined reconstruction of both ligaments versus isolated reconstruction of either of them. Combined MPTL and MPFL reconstruction may improve the outcomes compared to isolated reconstructions of either of them and/or decrease the need for other procedures, such as tibial tuberosity osteotomies (TTO), reducing surgical morbidity [24].

Historically, probably the first technique to surgically manage patellar instability was the Galeazzi procedure described in 1922, using a semitendinosus (ST) patellar tenodesis [25]. This procedure continued to be performed even in 1998, when the precise anatomical location of the insertion of MPFL was reported [20]. Rillmann et al. [20] described a transfer of the medial portion of the patellar tendon (PT). Both surgical techniques are analogous to a MPTL reconstruction. Many other authors reported their results with these techniques, with or without the reconstruction of the MPFL (e.g. lateral retinaculum release, TTO, Roux–Goldthwait) [26–28]. In the early 2000s, following the introduction of the isolated MPFL reconstruction, MPTL reconstruction fell out of favour. More recently, however, combined MPFL and MPTL reconstruction has been reported [13, 14, 22–24, 29–32].

An adequate tensile strength and length of the graft are the most important features for an ideal graft for combined reconstruction. In addition, the graft should have similar stiffness compared to the original ligaments to be reconstructed. The most commonly used grafts for ligaments reconstruction in the knee include the quadriceps, patellar, semitendinosus and gracilis tendons: all provide adequate strength in reconstruction procedures. However, because the MPTL is stiffer than the MPFL, the use of a stiffer graft for the MPTL than for the MPFL can be considered [11].

The purpose of this PRISMA compliant systematic review is to report techniques and clinical outcomes of reconstruction of the MPTL in combination with MPFL reconstruction in patients with lateral PF instability.

## Methods

**Fig. 1:**
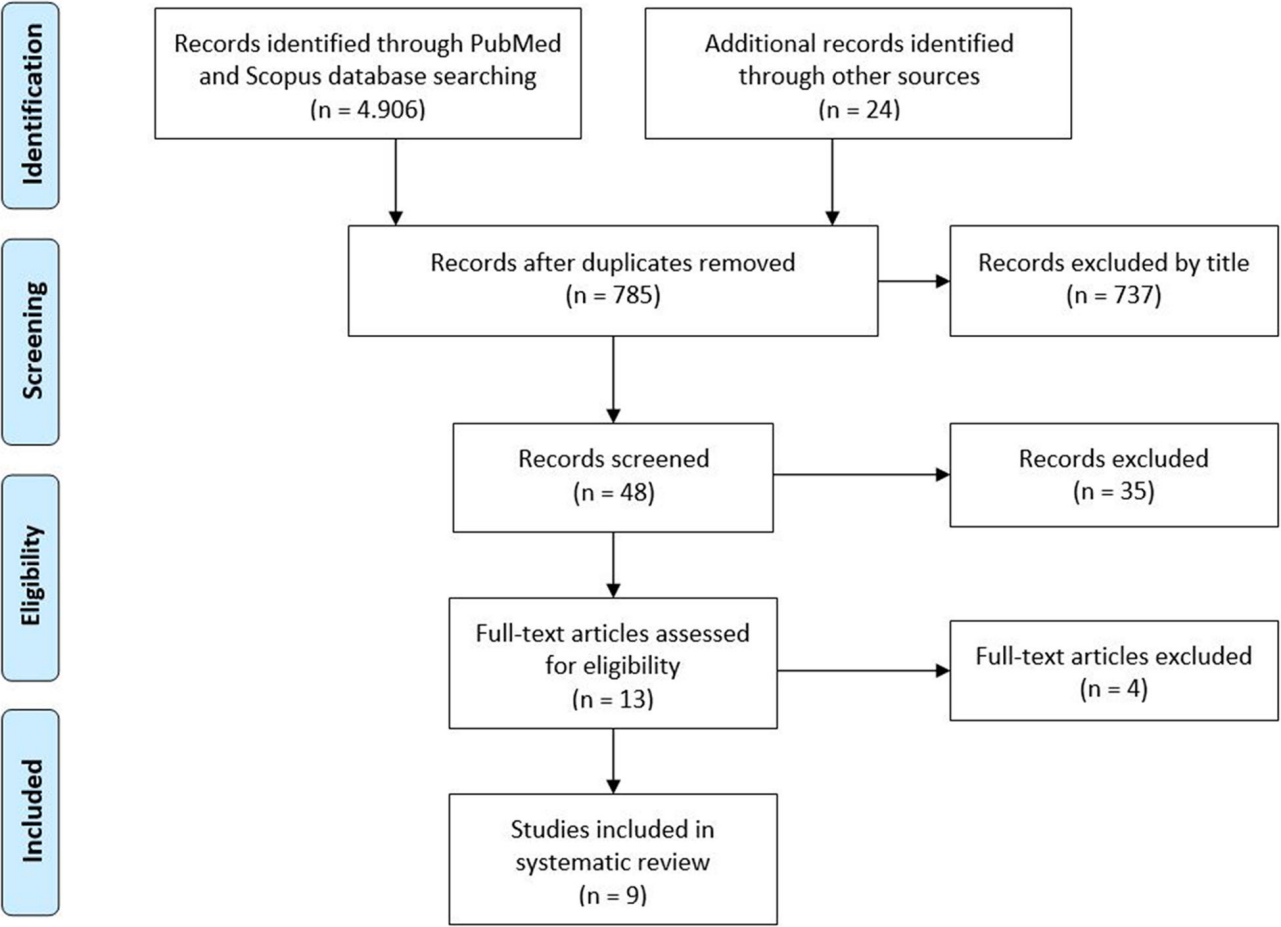
PRISMA flow diagram

This systematic review and its procedures were organized, conducted and reported following the Preferred Reporting Items for Systematic Reviews and Meta-Analyses (PRISMA) guidelines [33–35]. The PRISMA checklist is presented in Fig. 1.

We performed a systematic search (up to August 2020) in the PubMed and Scopus electronic databases to identify the available scientific articles about techniques and clinical outcomes of reconstruction of MPTL in combination with the MPFL in patients with lateral PF instability, with no restrictions of time and language.

For the purposes of our systematic review, we used several combinations of the following keywords: MPTL, MPFL, medial patellotibial ligament reconstruction, medial patellofemoral ligament reconstruction and patellar dislocation reconstruction. Editorials, technical notes, letters to authors, narrative reviews, systematic review articles and articles that did not report any clinical outcomes were excluded. Eligible articles could have been published in English, French, Italian, Spanish and Portuguese.

An orthopaedic resident (RA) performed the search and evaluated the articles. An experienced researcher in systematic reviews (NM) solved cases of doubt. At the beginning of the procedure, the investigator read the abstracts of all the articles, selected the relevant ones according to both inclusion and exclusion criteria and then compared the results with the other investigator. After 1 week, the same studies were read again to confirm the investigators’ agreement regarding articles selected. No disagreement was observed among the investigators.

The Coleman Methodology Score (CMS) was used to evaluate the quality of the articles included in this PRISMA systematic review [36]. The two authors (RA and NM) applied independently the CMS, and a final score was reached by consensus. The CMS is calculated by adding ten different criteria (study size, follow-up, number of procedures, type of study, diagnostic certainty, description of surgical technique, rehabilitation and compliance, outcome criteria, outcome assessment and selection process), with a maximum total possible score of 100 [36].

One investigator extracted the data from the full-text articles to Excel spreadsheet structured tables to analyse each study in a descriptive fashion. Another investigator independently double-checked the extraction of primary data from all the articles. Doubts and inconsistencies were grouped and solved. The information extracted from the articles is listed in Table 1.

**Table 1:** Clinical results of MPTL and MPFL reconstructions

References	***N***	Follow-up (mean)	Indications	Graft/technique	Associated risk factors	Results
Ebied and El-Kholy [12]	25	34 months	- At least 1 dislocation or subluxation - Persistent symptoms of instability despite 2 weeks of rehabilitation	ST and GT with distal insertion maintained One tunnel in the patella Lateral retinaculum release in 68% TTO in 32%	Trochlear dysplasia with: - Mean sulcus angle 141° Patella alta: not mentioned Lateral quadriceps vector: mean TTTG 15 mm	**Results**: 76% excellent, 20% good, and 4% fair **IKDC** scores: 54–81 **Effusion of the knee**: 8%
Brown and Ahmad [13]	2	14 months	Dislocation with normal osseous anatomy and mechanical alignment	ST and GT Distal insertion maintained Docking tunnel in the patella	No risk factors	**Redislocation**: none **Kujala scores**: 43–88 in 1 patient; 50–98 in 2 patients **Lysholm scores**: 38–97 in 1 patient 1; 55–95 in 2 patients
Sobhy et al. [20]	29	32.2 months	Recurrent dislocation with normal patellofemoral bone morphology and limb alignment with no other ligamentous deficiencies	ST and GT with distal insertion maintained Two tunnels in the patella	No risk factors	**Redislocation**: none **Returned to previous level of activity**:96.4% **Kujala scores**: 36.6–90.6 **Lysholm scores**: 51.9–89.5 **VAS** mean 6.3–1.8 **Cincinnati scores**: 50–88 **Congruence angle**: 11.93°–6.48° **Patellar tilt**: 10.9°–2.45° **Subjective instability**: 6.9%
Hinckel et al. [22]	7	5.5 months	Recurrent patellar dislocation with: - Subluxation in extension - Instability in flexion - Hyperextension of the knee with ligament laxity - Open growth plate with predisposing factors (increased lateral quadriceps vector, patella alta and trochlear dysplasia)	Medial quadriceps for the MPFL and medial patellar tendon for the MPTL Lateral retinaculum release in 42.9%. Shortening of the patellar tendon in 14.3%	Trochlear dysplasia with: - Modified Dejour classification: A (57.1%), B (14.3%), C (14.3%) and D (14.3%) Patella alta: CD > 1.2 (71.4%) Lateral quadriceps vector: TTTG > 20 mm (42.9%)	**Lengthening of the quadriceps tendon:** 14.3% **Redislocation**: none **Satisfaction** 9/10: 71.4% **Wound dehiscence**: 14.3%
Drez et al. [27]	15	31.5 months	- Recurrent instability after failed non-operative measures - Patients with patellar instability and a loose osteochondral fragment following patellar dislocation	ST (6 knees), ST + GT (5 knees), and Iliotibial band (3 knees). All as free grafts, one repair	- Trochlear dysplasia: not mentioned - Patella alta: normal - Lateral quadriceps vector: Q angle < 15°	**Redislocation**: none **Results**: 93% excellent and good. **Fulkerson score mean**: 93. **Kujala score** mean: 88.6. **Congruence angle**: 25.3°–5.5°. **Arthrofibrosis**: 6.7%. **Quadriceps atrophy**: 60%.
Yang and Zhang [28]	58	24 months	- At least two lateral patellar dislocations - Failure of a nonoperative treatment programme - 18 years at the time of surgery	ST with distal insertion maintained and fixed to tibial periosteum Proximal end fixed at the origin of the naïve MPFL in the medial aspect of the femur	- TT–TG distance > 20 mm - Grade of trochlear dysplasia - Patella alta: by Insall–Salvati ratio criteria	**Results**: 87.9% excellent, 6.9% good, 3.4% fair, 1.7% poor. **Kujala score**: 89.5 ± 10.2 **IKDC score**: 85 ± 13.9 **VAS**: 11 ± 4 **Patellar tilt**: 113 ± 5.2 **Insall–Salvati ratio**: 1.37 ± 0.19 **Modified Insall–Salvati ratio**: 1.95 ± 0.25 **TT–TG distance**: 19.9 ± 1.7 **Caton–Deschamps Index**: 1.31 ± 0.17
Sadigursky et al. [29]	7	12 months	- More than two episodes of patellar dislocation - MRI demonstrating extensive rupture of the medial retinaculum were included	ST with distal insertion maintained fixed with a metallic anchor inserted into the tibia The graft is percutaneously transferred to medial edge of the patella and fixated by a metal anchor And passed to the femur it is fixed by the fourth anchor in the Schöttle point	- Patellar height: Caton-Deschamps - Trochlear dysplasia - TT–TG distance	**Kujala score**: 88.57 ± 5.09 **Lysholm score:** 87.71 ± 5.70
Hetsroni et al. [30]	20	43 months	- History of recurrent lateral patellar instability - Physis closure - Patella which could be dislocable under anaesthesia	GT or ST with distal insertion maintained. Docking tunnel in the patella. it is fixed by the anchor in a mid-point between medial epicondyle and adductor tubercle	- BMI - Beighton score - TT–TG distance in the range (10–18 mm) - no cases of significant trochlea dysplasia or patella alta	**Kujala score**: 86.4 ± 12.5 **Tegner score**: 4.8 ± 2.4
Maffulli et al. [37]	34	3.1 years	- Two documented episodes of unilateral patellar dislocation necessitating reduction confirmed radiographically. - All patients undertaken intensive rehabilitation for 3 to 6 months after each documented episode of dislocation	Combined reconstruction of MPFL and MPTL using an ipsilateral autologous GT, passed into two patella tunnels, looped and sutured on the adductors magnus tendon	No risk factors	**Cincinnati Score i**ncrease to 90 ± 19 (*p* < 0.001) **Kajala score** increase to 82 ± 17 (*p* < 0.02) **Insall-Salvati index** remain within normal range No difference between males and females No difference with or without osteochondral lesions

## Results and discussion

After the initial literature search, a total of 4906 potentially relevant citations were identified. After removal of duplicate records, 785 articles were identified. After a first check of titles and abstracts, 737 articles were not included, since they did not investigate the outcomes of reconstruction of the MPTL in combination with the MPFL in patients with lateral PF instability. After further screening, other 39 articles were excluded since they did not conform to the inclusion criteria. A total of 9 articles (Table 1) were included in the present systematic review. The study selection process is reported in the PRISMA flowchart (Fig. 1). No randomized control trials were identified in our search, and all articles are case reports or case series.

Results of the CMS are reported in Table 2. There was a large range of CMS values, from 38 to 84, with a mean of 70.6 ± 14.4. Some of the selected studies reported a relatively small cohort of patients; had short follow-up, unclear outcome criteria and assessments and poor patient selection processes; and were retrospective.

**Table 2:** Results of the Coleman Methodology Score (CMS) used to assess the quality of the articles included

References	Study size	Follow-up	N procedures	Type of study	Diagnostic certainty	Description of surgical technique	Rehabilitation and compliance	Outcome criteria	Outcome assessment	Selection process	Total
Ebied and El-Kholy [12]	4	5	0	0	5	5	10	10	11	15	65
Brown and Ahmad [13]	0	2	10	0	5	5	0	10	6	0	38
Sobhy et al. [20]	4	5	10	10	5	5	10	10	10	15	84
Hinckel et al. [22]	0	0	10	10	5	5	10	10	5	15	70
Drez et al. [27]	0	5	10	0	5	5	10	10	6	13	64
Sadigursky et al. [29]	0	5	10	10	5	5	10	10	7	13	75
Hetsroni et al. [30]	4	5	10	10	5	5	10	10	10	15	84
Yang and Zhang [28]	7	2	10	0	5	5	10	10	10	15	74
Maffulli et al. [37]	4	5	10	10	5	5	10	10	10	13	82
**Maximum score possible**	10	5	10	15	5	5	10	10	15	15	100
**Mean ± standard deviation**	2.5 ± 2.6	3.7 ± 1.9	8.8 ± 3.3	5.5 ± 5.2	5 ± 0	5 ± 0	8.8 ± 3.3	10 ± 0	8.3 ± 2.2	12.6 ± 4.8	70.6 ± 14.4

In the analysed studies, most authors used hamstrings autografts, such as the gracilis tendon [37], preserving their tibial attachment [13, 14, 22, 30, 32]. In particular, two had free ends fixed in the tibia [24, 29, 31], eight used hamstrings [13, 14, 22, 29–32, 37], and one used the medial portion of the patellar and quadriceps tendons using anchors to fix the grafts in femoral and tibial attachments [24].

Furthermore, in three studies femoral tunnel fixation was undertaken using interference screws, with the proximal end of the hamstring graft employed to reconstruct the MPFL [13, 22, 30], in three other studies anchors were used to fix the semitendinosus grafts to the femur [29, 31, 32], while in one study the free end of the graft was looped and sutured to the adductor magnus tendon [37].

In this systematic review, we found many different techniques with wide variation in the graft choice, harvesting and fixation. The lengths of the various graft were different: 8.8 ± 8.4 cm for the quadriceps tendon [38], 4.9 cm for the patellar tendon [39], 36.6 cm for the semitendinosus tendon [39] and 41.9 cm for the gracilis tendon [39]. All grafts were long enough to reconstruct the MPFL (mean length of 60.6 mm) and MPTL (mean length of 36.4 mm) [7].

The tensile strength values were the following: for the quadriceps tendon, 1 cm in diameter of the superficial layer (stiffness of 33.6 ± 6.8 N/mm; yield load of 147.1 ± 65.1 N, maximum load to failure of 205 ± 77.8 N) [40]; for the medial third of the patellar tendon, 2734 ± 298 N [39]; for the semitendinosus tendon, 1216 ± 50 N [39]; and for the gracilis tendon, 838 ± 30 N [39].

The complications reported included wound infections [29, 41], quadriceps atrophy and subjective instability complaints [32], limitation of the range of motion (ROM) [22, 29] and effusion of the knee [13]. Three studies reported no complications in a total of 67 patients [14, 30, 31]. Wound complications, in a recent systematic review, were 11.9% overall, which may be related to extensive exposure and releases performed [42].

The present work identified a low number of articles that meet inclusion criteria (*N* = 9), with a relatively small number of operated knees (*N* = 197), reporting eight different techniques, all producing favourable outcomes with low rates of redislocation. The quality of articles is variable, from low to high.

Management of patellar instability with combined reconstruction of the MPFL and MPTL lacks of level I evidence which compares surgical techniques and biomechanical principles behind the mentioned techniques. Bitar et al. [43] compared operative and nonoperative management for recurrent patellar instability, with better subjective outcome after surgical treatment with MPFL reconstruction. The use of femoral soft-tissue fixation for MPFL reconstruction may reduce surgical morbidity but could be inferior in relation to patellar stability and patellar tracking; furthermore, it could result in an inferior clinical outcome compared with bone fixation of the graft. A recent RCT confirms that soft-tissue graft fixation did not result in an inferior subjective clinical outcome compared with screw fixation. Also, surgical morbidity at the femoral condyles was similar to those associated with screw fixation, with both techniques associated with an 11% incidence of significant pain at the femoral condyle with excellent patellar stability [44].

Generally, in the reported articles, patients had no additional risk factors for patellar instability. However, Ebied and El-Kholy performed a TTO in 32% (*N* = 8 of 25) of patients to correct a large quadriceps vector [13]. Hinckel et al. [24], in a study of 7 patients, reported a high-grade of trochlear dysplasia in 3 patients (43%), patella alta in 5 patients (71%) and a large quadriceps vector in 3 patients (43%), but only in one patient (14%), the patellar tendon was shortened.

Combined reconstruction of MPFL and MPTL is receiving increasing interest, probably due to new anatomical, biomechanical and histological studies which showed that the MPTL is a true ligament, with specific biomechanical proprieties important for patellofemoral tracking and stability [11, 45]. Probably, combined reconstruction may improve outcomes if compared with isolated MPFL reconstruction [13, 14, 22, 29], reducing not only surgical morbidity decreasing the need of bony procedures such as TTO in patients with borderline patella alta/lateralized force vector, but also the use of trochleoplasty in patients with moderate dysplasia [41].

Rehabilitation protocols are different and vary from restrictive (progressive weight-bearing with a brace locked in extension for 2 weeks, then ROM gradually increases in 30° increments every 2 weeks, and after 8 weeks the brace is discontinued and full weight-bearing and full range of motion are allowed) [22], to partially restrictive (full weight-bearing with crutches and isometric quadriceps strengthening, progressive increase of ROM to 0°–30° for the first 2 weeks, to 0°–90° for the fifth and sixth weeks, when the brace is removed and ROM allowed without restrictions) [32].

In the future, routine reconstruction of both MPFL and MPTL may become a part of the algorithms used for the management of patellar instability. However, as a meta-analysis was not possible, we can only conclude that good clinical outcomes were achieved by combined MPFL and MPTL reconstruction. In any case, it should be considered that most patients do well with isolated reconstruction of the MPFL [46–48], and it is not clear when a reconstruction of the MPTL should be added.

Given the lack of randomized controlled trials and the low number of studies, we are unable to define the ideal situation for combined MPTL and MPFL reconstruction. Probably, the indications suggested by Hinckel et al. [24, 49] (subluxation in extension, instability in flexion, knee hyperextension with ligamentous laxity, and skeletal immaturity with associated risk factors), supported by anatomical and biomechanical studies, may be used, at least until stronger clinical evidence is available.

The modified Coleman score [36] shows, on average, that the studies were of moderate quality, and the greatest limitation of this systematic review probably lies in the design of the reported studies. There are no randomized controlled trials comparing reconstruction of the MPFL and MPTL versus non-operative treatment, or comparing different operative treatments, and no article included a control group. Given the heterogeneity and small size of the cohorts studied, and the lack of randomized trials, a meta-analysis could not be performed.

A major strength of the present systematic review is the strict adherence to the PRISMA protocol and the use of accurate inclusion and exclusion criteria, which made our study reliable, since all the most up-to-date scientific evidence about the topic were meticulously examined.

Over the last few years, there has been increasing interest to the basic science and anatomic reconstruction of the MPFL and MPTL. At present, there is no clear consensus regarding the best technique to reconstruct the MPFL, and combined reconstruction of the MPTL and the MPFL could be effective in restoring patellar stability in patients with recurrent patella-femoral dislocation. We do not know whether such combined reconstruction would improve the outcomes relative to isolated MPFL or MPTL reconstructions, possibly decreasing the need for correction of other risk factors by osteotomies, thereby reducing surgical morbidity. We are aware that this can only be tested by appropriately powered randomized controlled trials.

Combined MPFL and MPTL reconstruction is safe and effective and allows surgeons to include these procedures in their surgical armamentarium for the management of recurrent patellar instability.

## Conclusions

The available scientific literature regarding combined MPTL and MPFL reconstruction suggests that this procedure leads to favourable clinical outcomes with minimal morbidity, supporting its use as a valid surgical alternative for the management of recurrent lateral patellar dislocations and clinical instability. However, the quality of the scientific articles available is variable, from low to high, and appropriately powered randomized controlled trials are needed to better understand what the adequate indications for surgery in case of patellar instability are.

## Data Availability

Not applicable

## Abbreviations

MPFL: Medial patellofemoral ligament
MPML: Medial patellomeniscal ligament
MPTL: Medial patellotibial ligament
CMS: Coleman Methodology Score
PF: Patellofemoral
TTO: Tibial tuberosity osteotomies
ST: Semitendinosus
GT: Gracilis tendon
PT: Patellar tendon
PRISMA: Preferred Reporting Items for Systematic Reviews and Meta-Analyses
ROM: Range of motion
